# Human Bocavirus in Korean Children with Gastroenteritis and Respiratory Tract Infections

**DOI:** 10.1155/2016/7507895

**Published:** 2016-11-20

**Authors:** Eun Jin Lee, Han-Sung Kim, Hyun Soo Kim, Jae-Seok Kim, Wonkeun Song, MiYoung Kim, Young Kyung Lee, Hee Jung Kang

**Affiliations:** Department of Laboratory Medicine, Hallym University College of Medicine, Gyeonggi-do, Republic of Korea

## Abstract

Human bocaviruses (HBoVs) are suggested to be etiologic agents of childhood respiratory and gastrointestinal infections. There are four main recognized genotypes of HBoVs (HBoV1–4); the HBoV-1 genotype is considered to be the primary etiologic agent in respiratory infections, whereas the HBoV2–4 genotypes have been mainly associated with gastrointestinal infections. The aim of the present study was to determine the distribution of HBoV genotypes in children with respiratory or gastrointestinal infections in a hospital in Korea. A total of 662 nasopharyngeal swabs (NPSs) and 155 fecal specimens were collected from children aged 5 years or less. Polymerase chain reaction (PCR) was conducted to detect the* NS1 *HBoV gene. The* VP1* gene of HBoV was further amplified in samples that were positive for the* NS1* gene. The PCR products of* VP1* gene amplification were genotyped by sequence analysis. HBoV was detected in 69 (14.5%) of 662 NPSs and in 10 (6.5%) of 155 fecal specimens. Thirty-three isolates from NPSs and five isolates from fecal specimens were genotyped, and all 38 sequenced isolates were identified as the HBoV-1 genotype. HBoV-1 is the most prevalent genotype in children with respiratory or gastrointestinal HBoV infections in a hospital in Korea.

## 1. Introduction

*Human bocavirus* (HBoV) belongs to the Parvoviridae family. Its classification as a bocavirus is based on similarities of the genetic structures and amino acid sequences with those of* Bovine parvovirus *and* Canine minute virus *[1–3]. Molecular biology studies have revealed that HBoV contains a 5.2-kb single-stranded DNA genome without an envelope [4]. The HBoV genome contains three open reading frames (ORFs), including the nonstructural protein-1 (*NS1*), nucleoprotein-1 (*NP1*), and viral capsid protein (*VP) 1/VP2 *genes, respectively [1, 5, 6]. NS1 is known to have regulatory functions, including transactivation or induction of apoptosis [7–9]. Although* NP1 *encodes nucleoproteins, its specific functions are unknown [9].* VP1/VP2 *encodes viral capsid proteins [2, 4]. Among these genes,* NS1* has the most conserved sequence, showing the lowest genetic diversity among all HBoV subtypes [10]; thus, this gene has been preferentially used as a target for the detection of HBoV [4, 11]. By contrast,* VP1/VP2 *forms a variable region showing high genetic diversity and has been mostly used for the phylogenetic analysis of HBoV [12, 13].

HBoV is a main cause of respiratory tract infection symptoms in infants and toddlers [4, 9, 14], whose manifestation varies from no symptoms to symptoms such as fever, coughing, and runny nose. In many cases, patients with HBoV detected in their fecal specimens present with symptoms of viral gastroenteritis, including diarrhea, vomiting, and fever [10].

There are four main HBoV genotypes, HBoV-1–4 [3, 6, 15]; HBoV-1 has been most frequently detected in respiratory specimens, whereas HBoV-2, HBoV-3, and HBoV-4 are most commonly found in fecal specimens, indicating a predisposition to causing gastrointestinal diseases [15–17]. However, there have been few studies on the association between gastroenteritis and HBoV isolates detected in fecal specimens.

The aim of this study was to investigate the detection rates of HBoV and its genotypes in respiratory specimens from children presenting with respiratory symptoms or in fecal specimens from children presenting with gastroenteritis symptoms in a tertiary hospital in South Korea.

## 2. Materials and Methods

### 2.1. Subjects

This study was conducted with two pediatric patient groups (under 6 years of age) with either gastroenteritis or respiratory tract infection symptoms. The gastroenteritis group included 155 fecal specimens obtained from pediatric patients who visited the Hallym University Sacred Heart Hospital between February 2015 and May 2015 and had undergone antigen tests for rotavirus, adenovirus, and norovirus. The respiratory tract infection group included 662 nasopharyngeal swabs (NPSs) obtained from pediatric patients who visited the Hallym University Sacred Heart Hospital while presenting with respiratory tract infection symptoms, including fever, coughing, and sputum during the same period of fecal specimen collection, and multiplex polymerase chain reactions (PCRs) for respiratory virus were requested. This study was approved by the Institutional Review Board/Ethics Committee of Hallym University Sacred Heart Hospital (IRB number 714203, 2014).

### 2.2. Test Methods

#### 2.2.1. Treatment of Specimens and Extraction of Nucleic Acids

Fecal specimens were mixed with phosphate-buffered saline (PBS) in 1 : 2-1 : 3 ratios and centrifuged at 6,000 ×g for 1 min. The obtained supernatant was used for the extraction of nucleic acids. NPSs were diluted in eNAT preservation solution (COPAN, Diagnostics, Murrieta, CA, USA) and directly used for the extraction of nucleic acids without pretreatment. Nucleic acids were extracted using a QIAamp Viral RNA Mini Kit (Qiagen, Hilden, Germany) via a QIACUBE device (Qiagen, Hilden, Germany) following the manufacturer's instructions.

#### 2.2.2. Detection of HBoV

HBoV was detected in fecal and respiratory specimens using PCR to identify the* NS1* gene [11]. PCR products were identified by electrophoresis using a Lab901 Screen Tape system (Agilent Technologies, Santa Clara, CA, USA).

#### 2.2.3. Nested PCR for HBoV

Stool specimens and NPSs that tested positive for the* NS1* gene via PCR were further subjected to nested PCR for amplification of the* VP1* gene, a variable region suitable for HBoV DNA sequence analysis [12].

#### 2.2.4. HBoV DNA Sequence Analysis and Identification of Genotypes

PCR products from amplification of the* VP1* gene of HBoV were subjected to DNA sequence analysis, and the resulting sequences were compared to13 DNA sequences of the four HBoV genotypes (HBoV-1–4) registered in the GenBank database (https://www.ncbi.nlm.nih.gov/genbank/) corresponding to the 3158–3520 nucleotide region of the* VP1* gene. The genotype distributions and relationships were compared based on the constructed phylogenetic tree. The DNA sequences were aligned using BioEdit software (ver. 7.2.5) and Clustal X (ver. 2.0). The phylogenetic tree was constructed using MEGA software (ver. 6.0), and the reliability was confirmed by 1,000 bootstrap replicates using the Hasegawa-Kishino-Yano algorithm. GenBank accession numbers of the DNA sequences used for the phylogenetic tree analysis of HBoV were as follows: EU984245, EF203920, EF450720, KJ634207.1, KC544963.1, EF690667 (HBoV1); FJ170280, FJ973558.1 (HBoV2A); FJ973560, FJ973559.1 (HBoV2B); EJ973563, FJ948861.1 (HBoV3); NC012729 (HBoV4).

## 3. Results

### 3.1. Detection of HBoV

**Figure 1:**
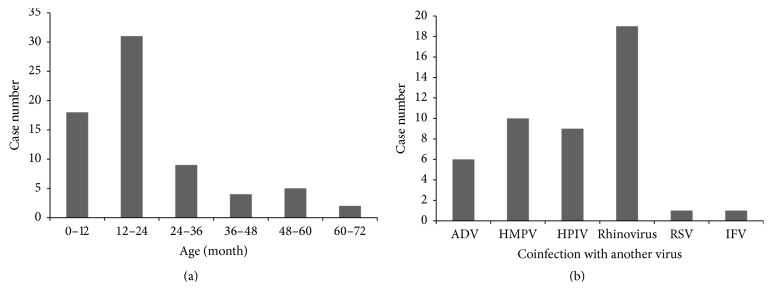
Distribution of children showing positive results in the detection of human bocavirus from nasopharyngeal swab specimens. (a) Age distribution (*N* = 69). (b) Coinfection with other viruses (*N* = 40). ADV: adenovirus; HMPV: human metapneumovirus; HPIV: human parainfluenza virus; RSV: respiratory syncytial virus; IFV: influenza virus.

Of the 155 fecal specimens, 10 specimens (6.5%, 7 males and 3 females) were positive for HBoV (Table 1). Of the 662 NPSs, 69 specimens (10.4%, 38 males and 31 females) were positive for HBoV. The mean and median age of the patients whose fecal specimens tested positive was 26.8 months and 14.5 months, respectively; the mean and median age of patients whose NPSs tested positive was 20.9 months and 17 months, respectively. Furthermore, 60% of the patients (6/10) whose fecal specimens tested positive and 71.0% of the patients (49/69) whose NPSs tested positive were 24 months old or younger (Table 1, Figure 1).

### 3.2. HBoV DNA Sequence Analysis and Identification of Genotypes

Of the 10 fecal specimens that tested positive in PCR for the* NS1 *gene, 7 cases (70%) also tested positive for the* VP1* gene with nested PCR. All 69 NPSs (100%) that tested positive for the* NS1* gene were also positive for the* VP1* gene.

**Figure 2:**
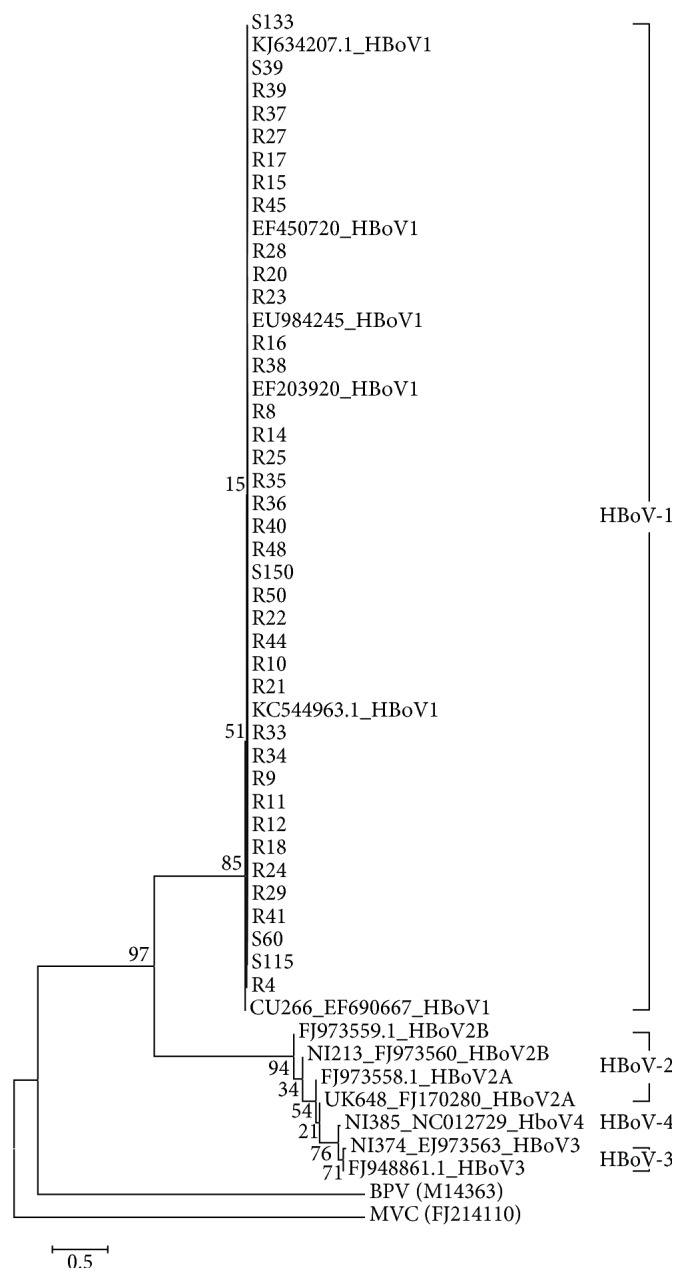
Phylogenetic tree constructed from the partial gene sequences encoding the HBoV polyprotein VP1. Virus names correspond to the country of origin/year collected/GenBank accession number. The scale bar represents a genetic distance of 0.1% (divergence in the nucleotide sequence). The phylogenetic tree was constructed using MEGA software (ver. 6.0), whose reliability was confirmed by 1,000 bootstrap replications using the Hasegawa-Kishino-Yano algorithm. F: fecal specimens; R: respiratory (nasopharyngeal) specimens, BPV:* Bovine parvovirus; *MVC:* Canine minute virus*.

Among the specimens testing positive for the* VP1* gene, 7 fecal specimens and 47 NPSs were subjected to DNA sequence analysis; 5 fecal specimens and 33 NPSs were suitable for DNA sequence analysis. The phylogenetic tree constructed on the basis of the DNA sequence analysis results including the 13 sequences from GenBank revealed two clades: HBoV-1 isolates formed the upper clade, and HBoV-2 (A and B), HBoV-3, and HBoV-4 isolates comprised the bottom clade. Consequently, the DNA sequences from all 38 analyzed cases (100%) were found to belong to HBoV-1, and the HBoV-2, HBoV-3, and HBoV-4 genotypes were not detected (Figure 2).

## 4. Discussion

In this study, the detection rates of HBoV were 6.5% in fecal specimens from pediatric patients with gastroenteritis and 10.4% in NPSs from children presenting with respiratory tract infection symptoms. Previous reports have indicated detection rates of HBoV in pediatric patients with respiratory tract infection symptoms ranging from 1.5% to 19% [9, 18, 19], and those in pediatric patients with gastroenteritis symptoms ranging from 0.8% to 42% [13, 20, 21]. Such high variation among studies may be due to differences in geography, season, and detection methods of HBoV. In particular, different studies used primers for the detection of HBoV that target different genes; however, the* NS1 *gene has been most commonly used in recent studies, because its ORF shows the lowest genetic diversity and it is highly conserved in all subtypes of HBoV [4]. Accordingly, in this study, we used primers specific for the* NS1 *gene to detect HBoV. Nevertheless, some fecal specimens in this study tested positive for the HBoV* NS1 *gene and were negative for the HBoV* VP1 *gene.

The prevalence of HBoV infections also varies depending on the season. Respiratory infection usually occurs from late spring to early summer [22], whereas gastroenteritis more frequently occurs in the winter and late spring [13]. The present study was conducted with specimens collected within a short period; therefore, one of the limitations of this study is that the overall incidence may have been biased based on the season of collection (late winter to spring).

Infants and toddlers are generally more prone to HBoV infection [4, 14]. Indeed, in this study, gastroenteritis and respiratory tract infection were more frequent in pediatric patients who were 2 years old or younger.

Previous studies have mostly detected HBoV-1 in respiratory specimens and HBoV-2a in fecal specimens [23]. However, HBoV-1 is also often detected in fecal specimens together with HBoV-2 [21], and in some regions, HBoV-1 was the most frequently detected HBoV genotype from fecal specimens [13, 24]. Although the etiology of gastroenteritis caused by HBoV-1 has not yet been clearly revealed, it has been speculated that HBoV first causes respiratory tract infections that persist without symptoms for several months; thereafter, the virus penetrates to the gastrointestinal tract and causes symptoms of gastroenteritis [21, 25]. In this study, only the HBoV-1 genotype was detected in both the NPSs and fecal specimens. This finding could support the hypothesis that HBoV in the respiratory tract is related to that in the gastrointestinal tract. However, no patient showed a positive result of HBoV in both NPSs and fecal specimens. This finding seems to be reasonable given that penetration of the virus from the respiratory tract to the gastrointestinal tract takes time [21, 25].

HBoV-2, HBoV-3, and HBoV-4 are rarely detected in the respiratory system [9, 16, 24]. Consistently, in the present phylogenetic analysis, HBoV-2, HBoV-3, and HBoV-4 were not detected in the NPS specimens. The primer used for DNA sequencing can detect all of the HBoV types (1, 2, 3, and 4), and a positive control was included for all four genotypes [12]. However, in this study, there was little genetic variability of HBoV among children with symptoms. This might be related to geographic factors, given that all of the children were residents of a specific area visiting a single institution.

The gastroenteritis group consisted of children who showed symptoms from a viral infection. However, a traditional etiologic viral agent (rotavirus, adenovirus, or norovirus) could not be confirmed in all fecal specimens. Given that HBoV was the only virus detected in the fecal specimens of seven patients, we could surmise that HBoV is a causative virus of gastroenteritis.

In conclusion, in this study, HBoV was detected in patients with respiratory tract infections as well as in those with gastroenteritis symptoms, suggesting HBoV as a causative virus for gastroenteritis. In addition, genotype analysis of the HBoV isolates detected in the study samples showed that all HBoV detected in children with gastroenteritis as well as those with respiratory tract infection were of the HBoV-1 genotype.

## Figures and Tables

**Table 1:** List of children showing positive results for the detection of human bocavirus in fecal specimens.

Specimen number	Age (month)	Coinfection with other viruses
S4	10	No coinfection
S6	17	No coinfection
S39	72	No coinfection
S60	1	No coinfection
S115	0.5	Rotavirus
S133	12	Norovirus
S135	60	Rotavirus
S141	48	No coinfection
S150	6	No coinfection
S155	42	No coinfection

## References

[1] Allander T., Tammi M. T., Eriksson M., Bjerkner A., Tiveljung-Lindell A., Andersson B. (2005). Cloning of a human parvovirus by molecular screening of respiratory tract samples. *Proceedings of the National Academy of Sciences of the United States of America*.

[2] McIntosh K. (2006). Human bocavirus: developing evidence for pathogenicity. *Journal of Infectious Diseases*.

[3] Kapoor A., Slikas E., Simmonds P. (2009). A newly identified bocavirus species in human stool. *Journal of Infectious Diseases*.

[4] Lindner J., Modrow S. (2008). Human bocavirus—a novel parvovirus to infect humans. *Intervirology*.

[5] Chen K. C., Shull B. C., Moses E. A., Lederman M., Stout E. R., Bates R. C. (1986). Complete nucleotide sequence and genome organization of bovine parvovirus. *Journal of Virology*.

[6] Cashman O., O'Shea H. (2012). Detection of human bocaviruses 1, 2 and 3 in Irish children presenting with gastroenteritis. *Archives of Virology*.

[7] Moffatt S., Yaegashi N., Tada K., Tanaka N., Sugamura K. (1998). Human parvovirus B19 nonstructural (NS1) protein induces apoptosis in erythroid lineage cells. *Journal of Virology*.

[8] Raab U., Beckenlehner K., Lowin T., Niller H.-H., Doyle S., Modrow S. (2002). NS1 protein of parvovirus B19 interacts directly with DNA sequences of the p6 promoter and with the cellular transcription factors Sp1/Sp3. *Virology*.

[9] Schildgen O., Müller A., Allander T. (2008). Human bocavirus: passenger or pathogen in acute respiratory tract infections?. *Clinical Microbiology Reviews*.

[10] Chieochansin T., Chutinimitkul S., Payungporn S. (2007). Complete coding sequences and phylogenetic analysis of human bocavirus (HBoV). *Virus Research*.

[11] Maggi F., Andreoli E., Pifferi M., Meschi S., Rocchi J., Bendinelli M. (2007). Human bocavirus in Italian patients with respiratory diseases. *Journal of Clinical Virology*.

[12] Blinkova O., Rosario K., Li L. (2009). Frequent detection of highly diverse variants of *Cardiovirus, Cosavirus, Bocavirus*, and *Circovirus* in sewage samples collected in the United States. *Journal of Clinical Microbiology*.

[13] Alam M. M., Khurshid A., Shaukat S. (2015). 'Human bocavirus in Pakistani children with gastroenteritis. *Journal of Medical Virology*.

[14] Arnold J. C., Singh K. K., Spector S. A., Sawyer M. H. (2006). Human bocavirus: prevalence and clinical spectrum at a children's hospital. *Clinical Infectious Diseases*.

[15] Albuquerque M. C. M., Rocha L. N., Benati F. J. (2007). Human bocavirus infection in children with gastroenteritis, Brazil. *Emerging Infectious Diseases*.

[16] Arthur J. L., Higgins G. D., Davidson G. P., Givney R. C., Ratcliff R. M. (2009). A novel bocavirus associated with acute gastroenteritis in Australian children. *PLoS Pathogens*.

[17] Kapoor A., Simmonds P., Slikas E. (2010). Human bocaviruses are highly diverse, dispersed, recombination prone, and prevalent in enteric infections. *Journal of Infectious Diseases*.

[18] Bastien N., Brandt K., Dust K., Ward D., Li Y. (2006). Human bocavirus infection, Canada. *Emerging Infectious Diseases*.

[19] Allander T., Jartti T., Gupta S. (2007). Human bocavirus and acute wheezing in children. *Clinical Infectious Diseases*.

[20] Lee J. I., Chung J. Y., Han T. H. (2007). Detection of human bocavirus in children hospitalized because of acute gastroenteritis. *Journal of Infectious Diseases*.

[21] Campos G. S., Silva Sampaio M. L., Menezes A. D. L. (2016). Human bocavirus in acute gastroenteritis in children in Brazil. *Journal of Medical Virology*.

[22] Choi E. H., Lee H. J., Kim S. J. (2006). The association of newly identified respiratory viruses with lower respiratory tract infections in Korean children, 2000–2005. *Clinical Infectious Diseases*.

[23] Han T.-H., Kim C.-H., Park S.-H., Kim E.-J., Chung J.-Y., Hwang E.-S. (2009). Detection of human bocavirus-2 in children with acute gastroenteritis in South Korea. *Archives of Virology*.

[24] Chow B. D. W., Ou Z., Esper F. P. (2010). Newly recognized bocaviruses (HBoV, HBoV2) in children and adults with gastrointestinal illness in the United States. *Journal of Clinical Virology*.

[25] Vicente D., Cilla G., Montes M., Pérez-Yarza E. G., Pérez-Trallero E. (2007). Human bocavirus, a respiratory and enteric virus. *Emerging Infectious Diseases*.

